# Method matters: impact of in-scenario instruction on simulation-based teamwork training

**DOI:** 10.1186/s41077-017-0059-9

**Published:** 2017-11-28

**Authors:** Cecilia Escher, Hans Rystedt, Johan Creutzfeldt, Lisbet Meurling, Sofia Nyström, Johanna Dahlberg, Samuel Edelbring, Torben Nordahl Amorøe, Håkan Hult, Li Felländer-Tsai, Madeleine Abrandt-Dahlgren

**Affiliations:** 1 CLINTEC–Department of Clinical Science Interventions and Technology, Karolinska Institutet, Stockholm, Sweden; 2 CAMST-Center for Advanced Medical Simulation and Training, Karolinska University Hospital, Stockholm, Sweden; 3 Department of Education, Communication and Learning, University of Gothenburg, Gothenburg, Sweden; 4 Department of Behavior Sciences and Learning, Linköping University, Linköping, Sweden; 5 Department of Clinical and Experimental Medicine, Linköping University, Linköping, Sweden; 6 Department of Medical and Health Sciences, Linköping University, Linköping, Sweden; 7 Department of Learning, Informatics, Management and Ethics, Karolinska Institutet, Stockholm, Sweden; 8 Simulator Centre West, Sahlgrenska University Hospital, Gothenburg, Sweden

**Keywords:** Simulation, Healthcare, Crew resource management, Interprofessional education, Instructor, Facilitator, Video analysis, Teamwork, Fidelity, Cueing

## Abstract

**Background:**

The rationale for introducing full-scale patient simulators in training to improve patient safety is to recreate clinical situations in a realistic setting. Although high-fidelity simulators mimic a wide range of human features, simulators differ from the body of a sick patient. The gap between the simulator and the human body implies a need for facilitators to provide information to help participants understand scenarios. The authors aimed at describing different methods that facilitators in our dataset used to provide such extra scenario information and how the different methods to convey information affected how scenarios played out.

**Methods:**

A descriptive qualitative study was conducted to examine the variation of methods to deliver extra scenario information to participants. A multistage approach was employed. The authors selected film clips from a shared database of 31 scenarios from three participating simulation centers. A multidisciplinary research team performed a collaborative analysis of representative film clips focusing on the interplay between participants, facilitators, and the physical environment. After that, the entire material was revisited to further examine and elaborate the initial findings.

**Results:**

The material displayed four distinct methods for facilitators to convey information to participants in simulation-based teamwork training. The choice of method had impact on the participating teams regarding flow of work, pace, and team communication. Facilitators’ close access to the teams’ activities when present in the simulation suite, either embodied or disembodied in the simulation, facilitated the timing for providing information, which was critical for maintaining the flow of activities in the scenario. The mediation of information by a loudspeaker or an earpiece from the adjacent operator room could be disturbing for team communication.

**Conclusions:**

In-scenario instruction is an essential component of simulation-based teamwork training that has been largely overlooked in previous research. The ways in which facilitators convey information about the simulated patient have the potential to shape the simulation activities and thereby serve different learning goals. Although immediate timing to maintain an adequate pace is necessary for professionals to engage in training of medical emergencies, novices may gain from a slower tempo to train complex clinical team tasks systematically.

## Background

The purpose of medical simulations is for students and professionals to practice skills in a safe environment without risk to patients and to transfer the lessons learned to clinical practice [1, 2]. The conditions for transfer rely on the assumption that there is a relevant resemblance between the simulation and the clinical task, often expressed in terms of fidelity. However, researchers have discussed fidelity in medical simulation since the first simulators were constructed, and the term has different meanings [3]. The definition of structural fidelity refers to the physical and physiological resemblance of the simulator and real-world features, while psychological fidelity is the sense of realism relative to the learning goals for the session [4–6]. In contrast, Hamstra et al. [7] suggest that the traditional concept of fidelity should be abandoned. The reason, they argue, is that higher fidelity regarding the physical properties of the simulator does not necessarily correlate with effective learning. Instead, they suggest the concept of functional task alignment, which refers to “the fidelity of simulation scenario relative the clinical task demands”^7(p388)^.

Instead of focusing on pre-defined conceptualization of fidelity, in this study we rely on empirical studies on how gaps between the simulation and the clinical task emerge and how such gaps can be bridged [8]. Simulation-based teamwork training (SBTT) can be particularly demanding regarding functional task alignment. Verbal communication from facilitators can easily interfere with team communication as messages from the facilitator are sent “on the same channel” as the team communication which is of particular importance to the training. As pointed out by Johnson [9], facilitators’ talk and gestures are essential for reconstituting simulations as a kind of medical practice for instructional purposes. From this idea, it follows that the simulations’ relevance for learning is highly dependent on the facilitators’ instructions. This, in turn, calls for further scrutiny of how gaps between the simulator and the clinical task can be bridged by facilitators.

Although high-fidelity simulators are highly interactive and complex, some features of the human body are not well represented (such as skin texture, skin color, facial expression, muscle tonus, and movements). Often, such features are basic for displaying how patients’ conditions develop and are prerequisites for training the management of acute conditions. To bridge the gap between the appearance of a sick patient and the mannequin, facilitators often supply participants with various forms of additional information, which we refer to as *extra scenario information*. A close notion is cueing, as suggested in a review by Paige et al. [10], referring to how instructors and features of the simulator itself could provide cues on how the scenario should be understood. In contrast, our conceptualization of extra scenario information refers to information exclusively conveyed by facilitators, not information as provided by the equipment or environment. Based upon the limited human signs the simulator can display, our interest is directed to how the educator help the participants understand the situation and act as if the simulator was a real patient [11]. For instance, facilitators may add information about bodily features, which in clinical work is perceived by hearing, vision, touch, and sometimes even smell, to give the participants an appropriate picture that makes an assessment and clinical decisions possible.

Several methods for delivering extra scenario information are available in simulation-based team training. One possibility for facilitators when simulating verbal patients is to convey information by simulating the patients´ voice through a loudspeaker in the mannequin, however, this method is impossible in scenarios simulating neonatal or unconscious patients. Another possibility described in the literature is a facilitator acting as a confederate, i.e., an actor or faculty member roleplaying in the scenario also being able to provide extra scenario information [12, 13]. The role of a confederate is complex and may require scripting and some acting skills from the facilitator [14]. Another option for facilitators is to provide extra scenario information from the operator room via a loudspeaker or via an earpiece to one of the participants in the scenario [15]. This means that facilitators have a very important role to help participants understand the scenario and to frame the simulation as a professionally relevant learning experience, a very different role from medical teaching in traditional settings [5, 9, 16]. Although such supplementary information is a well-known component of almost all simulation-based teamwork training, scholars have not systematically studied the implications of different methods to provide extra scenario information regarding what they offer in a team training context.

On this background, a descriptive qualitative study was conducted by analyzing a set of video recordings from different simulation-based teamwork training contexts. Our aim was to identify and describe facilitators´ various methods for providing extra scenario information in our dataset, what occasioned such information and its consequences for the participants’ interaction. These findings, in turn, form the basis for discussing the educational implications of the observed methods to provide extra scenario information.

More specifically, we address the following research questions:

1. What characterize the observed methods to convey extra scenario information?
2. What triggers facilitators to provide extra scenario information?
3. What visible impact do the methods for providing extra scenario information have on participants’ activities in the scenario?

## Methods

The study is part of a multicenter research project on interprofessional simulation-based education in which the researchers analyzed video-recorded scenarios from three well-established academic Swedish simulation centers. All simulations were performed in simulation studios during regular scheduled courses. The method used is descriptive qualitative in order to capture interactions between participants, facilitators, and the simulator. The study is exploratory, as methods to convey extra scenario information have not yet been systematically studied.

The research team consisted of seven physicians with extensive experience in education and research on medical simulation, four senior researchers in medical education who specialize in interprofessional education, two senior researchers who specialize in educational science, and an experienced psychologist and educator. In accordance with the project plan focus for the analysis was proposed by CE and LM but further defined before the analysis commenced.

### Material

In this study, we sampled 2- to 4-min film clips of SBTT from a shared database of 31 scenarios. The selected scenarios included simulation-based team training on resuscitation of a neonate, trauma resuscitation in the emergency department, and the management of a medical emergency in a hospital ward and at a health center. The participants in the resuscitation of the neonate scenario and some of the emergency department scenarios were professionals; the participants in the other scenarios were nursing and medical students. Participants were not homogenous regarding prior exposure to simulation-based education. The facilitators had basic but varying levels of knowledge and experience regarding healthcare education and simulation.

### Data analysis

The analysis is based on foundational principles for qualitative video analysis in the social sciences as suggested by Heath et al. (2010) [17], implying a focus on the interplay between participants and the physical environment. This means that *interaction* constitutes the unit of analysis, i.e., how facilitators and trainees in the simulation act and respond to each other’s talk and actions in the simulation. The rationale for using video is to preserve details of the interaction—such as talk, gestures, and positioning—enabling a close scrutiny of how facilitators conveyed information, how it was triggered, and its implications on the participants’ actions. Further, data can be revisited in collaborative analyses, which in this study comprised three phases [18]. In the first phase, two of the authors, CE and LM, identified episodes in the 31 scenarios in which the facilitators intervened by providing extra scenario information. Out of these, a set of five film clips (2–4 min each) were selected that were aimed to capture a variation of methods for providing extra scenario information. This sample, in turn, provided the basis for a 1-day multidisciplinary analysis [18]. During this phase, each video clip was observed by two subgroups while taking field notes that provided the basis for discussion in the whole group. The purpose was to develop a collectively enriched and shared understanding of the observed sequence of activities in each scenario and to ensure the empirical anchoring of the initial findings. The results of the analysis were summarized on a shared screen to assure consensus in the research group. In the third phase, the five film clips were transcribed and CE, together with HR and JC, revisited the film clips, transcripts, and documentation from the collaborative analysis. In this last phase, all 31 scenarios were revisited to anchor the finding in the entire data material by identifying both variations and deviant cases in relation to the initial findings [17].

## Results

The facilitators at all centers supplied extra scenario information regarding, for example, skin color, neurological, and abdominal assessment, but there were significant differences in the focus of this information and the way the facilitator conveyed it. We identified four methods for providing extra scenario information. Further, we found systematic differences between the methods regarding how the need for extra scenario information was occasioned. The facilitators delivered information either as a response to participants’ questions or in response to their action. Most important, the different methods of delivering information affected the pace, workflow, and interplay between the participants of the team.

### Method 1: information via a confederate

**Fig. 1:**
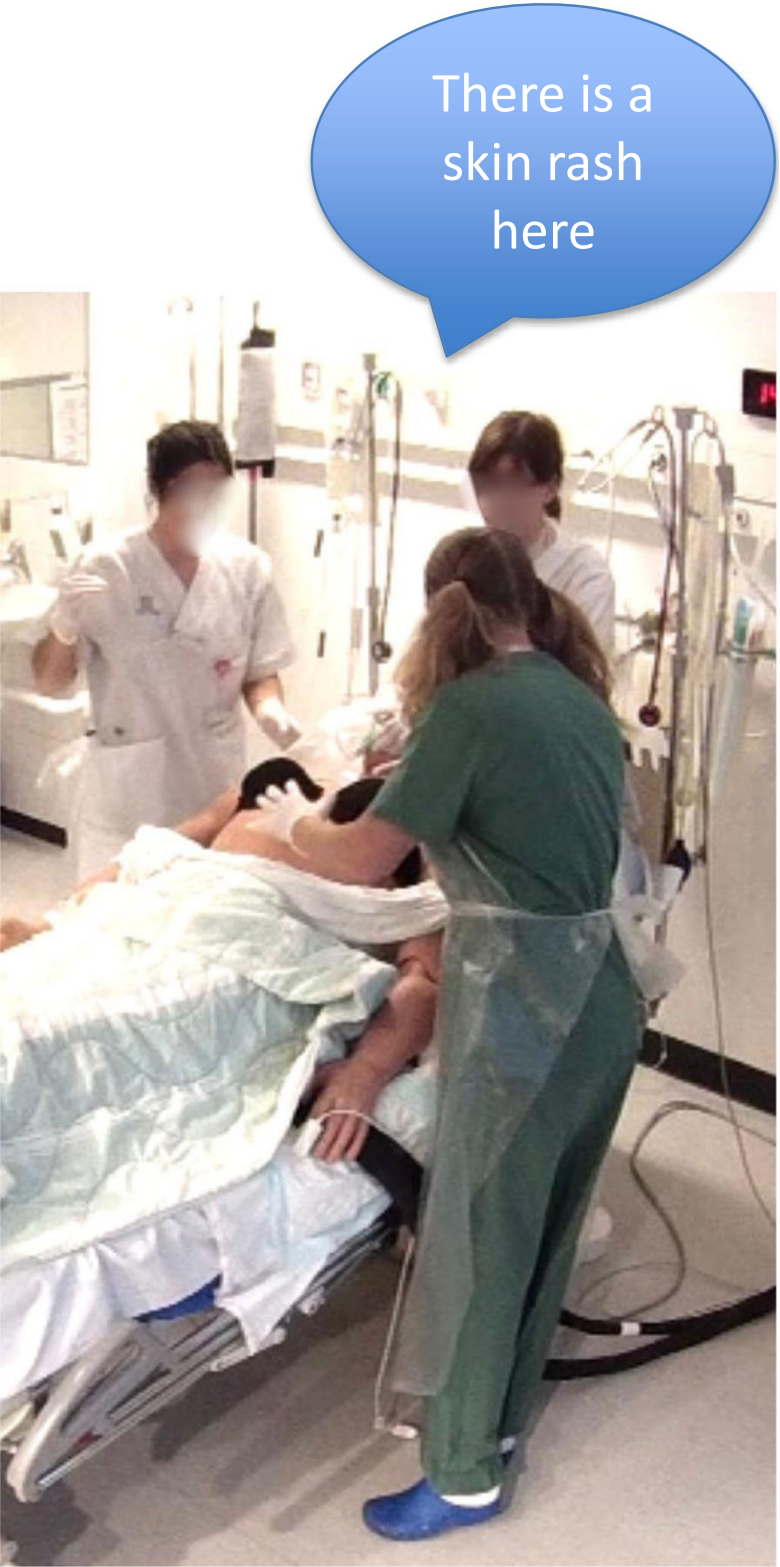
Facilitator giving information regarding a skin rash using verbal and body language

This method was found in 10 hospital emergency scenarios for medical students as well as in interprofessional team training for staff. Facilitators who assisted in the simulator studio followed the scenarios closely and responded promptly to the participants’ actions (such as examining the skin) and questions. The entire team could see and hear the facilitator adding information. At times, information was not only delivered non-verbally, for example, by showing the location of a skin rash on the patient’s chest (Fig. 1) but also verbally by adding details about the presence or absence of pathological signs that the simulator did not display. The facilitators moved in and out of the team. By remaining distant, they allowed room for the participants to take the initiative, which in turn, formed the basis for further instructions. By moving closer, the facilitators got immediate access to details of the participants’ actions and could respond promptly. The close access to the activities in the scenario enabled the facilitator to anticipate the next step without interfering with the pace and the flow of the work. This enabled team members to mainly communicate with each other and to keep focus on the evolving problem. By using prompting questions, the facilitators also helped participants to move on in the problem-solving process, for example, by asking, “You want to give medication? What do you suspect?”

### Method 2: information via a bystander

**Fig. 2:**
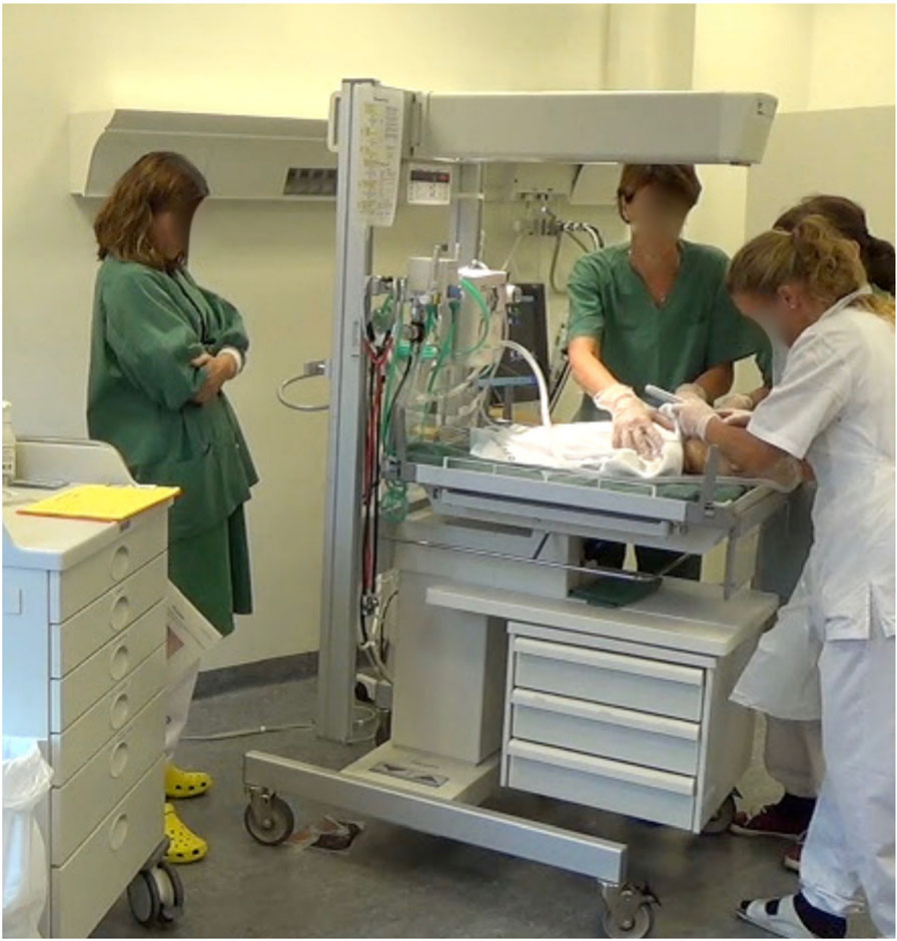
Facilitator staying close to the team, not participating in the scenario

This method was used in three scenarios of neonatal resuscitation training for interprofessional teams of experienced staff. The facilitators took position at the head end of the resuscitation table, very close to the team. The facilitators clearly displayed that they were not expected to take part in the team work, for instance, by remaining in the same position and keeping arms crossed the whole time (Fig. 2). This stance enabled the facilitator to follow the scenario closely and to deliver timely instructions in relation to how the scenario developed and to deliver requested information that the entire team could hear. The facilitators, at times assisted by a written script, used professional jargon, rich with metaphors such as “the meconium is as thick as pea soup”. Some of the information given was provided in terms of an algorithm, such as “color zero”, referring to the skin color of the infant translated into an Apgar score. Speedy talk, short messages, and coded information maintained a fast work pace. The mannequin representing the neonate displayed only a few significant signs for the scenario, neither muscle tonus, skin color, or voice. Although the facilitator delivered a lot of extra scenario information, the communication remained mainly among the team members who stayed focused on the task. The messages were brief and timely to correspond to the need for immediate and prompt action in the resuscitation of a neonate.

### Method 3: information via a loudspeaker

**Fig. 3:**
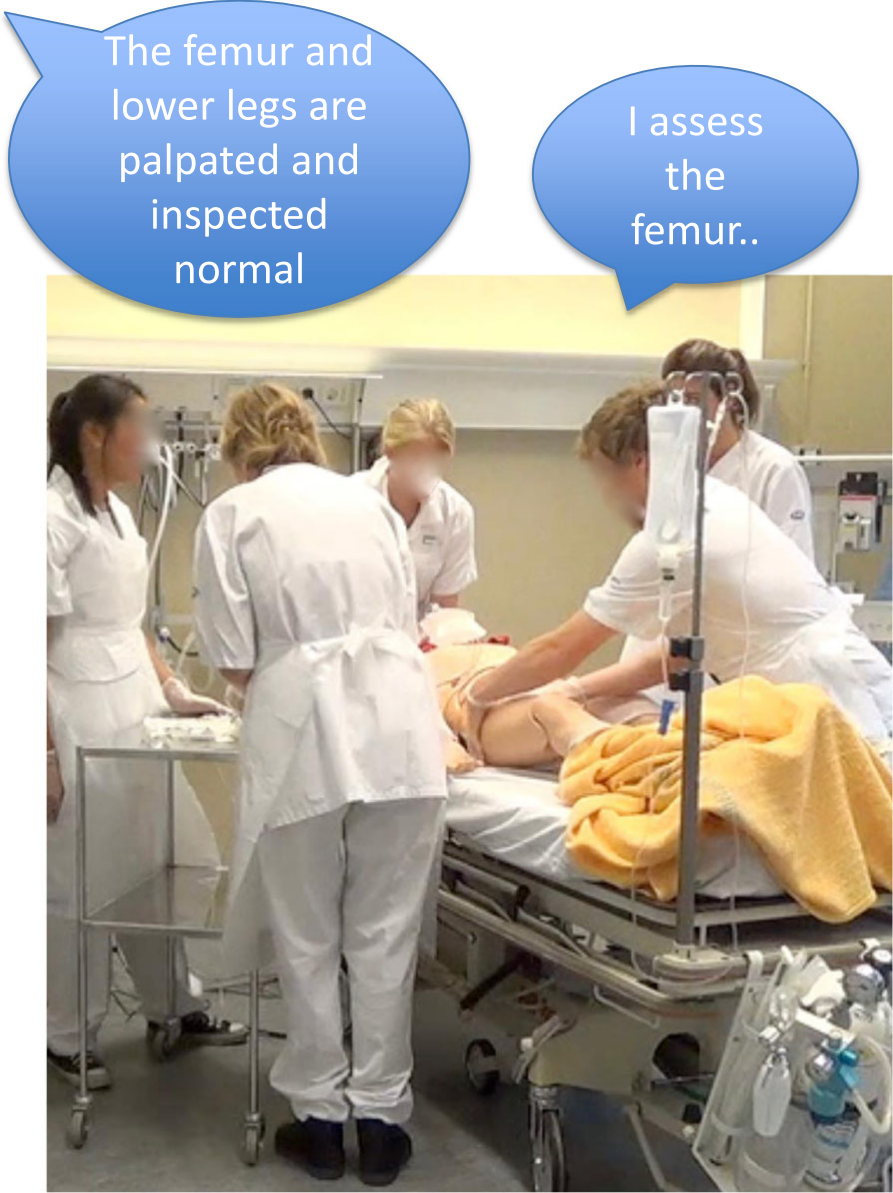
The team leader speaking out actions to get information from the facilitator situated in an adjacent operator room

This method was used in eight trauma scenarios in which nursing and medical students trained interprofessional teamwork. A facilitator in the adjacent operator room, overseeing the training through a one-way screen, delivered information concerning the findings via a loudspeaker. In addition, another facilitator was present in the studio to help out with practicalities, but (except for a few instances) without providing extra scenario information or intervening in the teamwork. Instructions from the operator room were heard by the entire group of students while performing the ABCDE algorithm and were at times given upon request from the participants. Long sentences that used formal medical terminology similar to medical records characterized the language that differed distinctly from naturally occurring speech. For example, when a student examined the patient’s legs and reported what he was doing, the facilitator answered, “lower extremities are inspected and palpated normal”. The speech was slow and recurrently occasioned the teams to stop and listen. Typically, the trauma team leader announced all actions when performing them one by one and then paused briefly to wait for further information from the facilitator via the loudspeaker (Fig. 3). The slow speed of the speech, the lengthy messages, and the delay in waiting for the next piece of information from the facilitator compromised the workflow and slowed down the pace. The communication at times mainly occurred between the team leader and the facilitator. When the team leader was occupied listening to information from the facilitator, information from team members did not get through, which impaired communication among the team members. In instances when the information given was brief and well timed, the communication occurred mainly within the team.

### Method 4: information via an earpiece

**Fig. 4:**
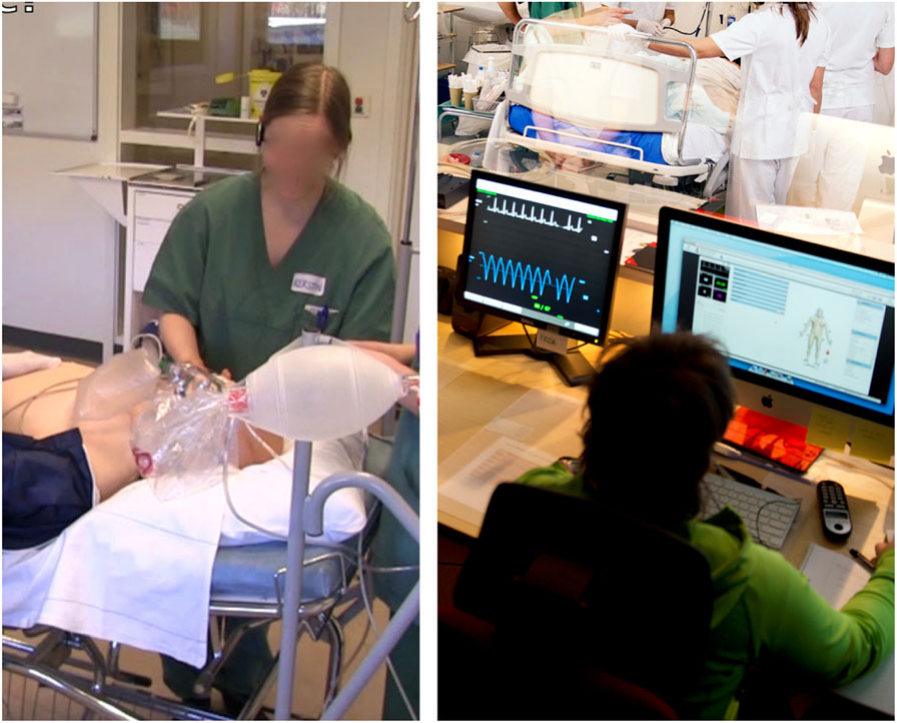
The participant wearing the earpiece receives information from the facilitator and conveys it to the team

This method was used in nine scenarios of emergency situations from an interprofessional training program for medical and nursing students. The facilitator was situated in the adjacent operator room and conveyed extra scenario information to one of the participants through an earpiece. The participant wearing the earpiece then forwarded the information to the rest of the team (Fig. 4). At times, the team members called out requests for additional information for example “what do the pupils look like?” Information regarding the setting was also conveyed via the earpiece, for example, one participant reported that “you can’t administer iv. antibiotics because this is a health center”. On these occasions, the receiving team member seemed to “step out of the scenario” to wait and focus on the information he or she received. In addition to participating in the tasks at hand and communicating within the emergency team, mediating extra scenario information as an extra task at times seemed to split the team member’s attention. Most of the communication took place between the team members; however, the team communication was temporarily disturbed when the participants asking for information and the participant wearing the earpiece listened to instructions from the operator room. However, some participants seemed to cope with the earpiece and convey information with little interruption of their own activities or the teamwork. In one scenario, the last one in a series of five during the 1 day training, no extra scenario information was conveyed via the earpiece at all. This seemed to be associated with the fact that the participants were familiar with the situation and managed to request and get relevant information from the facilitator via the simulators voice.

## Discussion

Although the mannequins used in healthcare simulations are highly sophisticated, the present results demonstrate that facilitators’ extra scenario information is essential for bridging the gap between the appearance of a sick patient and a human patient simulator. We argue that this information is important for participants to learn through iterative processes of assessments and actions. It is crucial to provide pieces of information, such as skin rash, thickness of the meconium, or abdominal status, for enabling the teams’ assessment and decision-making.

We agree, as proposed by Paige et al. [10], that conceptualization of fidelity should be extended by including various forms of cueing and the needs for empirical research on this aspect of simulation practice. Although our study solely focuses on the facilitators’ role, it provides further arguments for shifting emphasis from the properties of the simulator per se to session design and facilitators’ actions to create simulation activities as relevant representations of the intended clinical task. With reference to Hamstra et al. [7], we argue that this could contribute to functional task alignment, by framing the scenario in accordance with the intended learning objectives. Without the extra information, the participants would not have responded to the simulations as instances of the intended problem, and there would have been insufficient signs for the participants to draw conclusions about how to continue. In line with Johnson [9], the facilitators contribute to reconstituting the simulation as a medical practice, which, in turn, can serve as a context for focusing on the relevant aspects of professional performance. However, this does not mean that the level of structural fidelity does not matter, as the properties of the simulator serve as a basis for what information facilitators provide. Moreover, the facilitators have a delicate task to transform visual and sensory signs regarding bodily attributes into gestures and verbal messages to ensure that the participants have enough clues to understand the case without interfering with their work. Our study brings attention to the intricate interplay between humans and technology and how facilitators continuously adapt to the simulators´ shortcomings in order to keep the momentum of the scenario and enhance learner engagement.

In relation to what occasioned the facilitators to give extra scenario information, we found that the different methods offered different opportunities for facilitators to assess the teams’ needs for information. When the facilitators were present in the room (methods 1 and 2), and positioned close to the team, they used the participants’ actions and the evolving condition of the imagined patient as the basis for delivering timely prompts and instructions. When the facilitator conveyed the information via a loudspeaker or an earpiece (methods 3 and 4), the participants’ verbal reports on their actions or explicit questions often occasioned the facilitators to respond. In most cases, the facilitator responded after a delay, and the response was not as closely aligned to the teams’ actions and the development of the scenario as when the facilitator was present in the room. However, practical limitations exist. The facilitator may be located in an adjacent operator room because, in addition to adding extra scenario information, he or she may be operating the simulator and acting as the patient’s voice. The complex tasks of educators in healthcare simulation [19, 20] and an increased workload using low-fidelity compared to high-fidelity mannequins imply that multitasking can also be a reason for less well-timed information [21].

The methods for providing extra scenario information had extensive implications for how the scenarios played out and the opportunities for teamwork training. First, we observed an impact on communication patterns. Methods 1 and 2, with a facilitator present close to the action, tended to promote horizontal communication (i.e., to maintain the communication between team members). Providing information through method 3 and 4 tended to promote vertical communication (i.e., between a team member and the facilitator). In our cases, the learning goals were to train non-technical skills, such as interprofessional communication and decision-making [2] in which lengthy verbal information from the facilitator that hampers the internal communication among the team members might be particularly unwanted. In short, vertical communication tended to impair horizontal communication and to counteract the intended learning objectives of the simulation.

Second, the methods of providing information affected the workflow. Timely information in the form of brief prompts in method 1 and 2 seldom disturbed the course of events. In contrast, information delivered through method 3 impeded workflow. When the facilitator delivered lengthy verbal information slowly, the team had to pause and listen to the response. In method 4, the activities stopped for brief moments when the team had to ask for essential information. In both cases, the pace of the facilitator’s response interrupted the flow of the teamwork. This disruption occurred regularly in method 3 and occasionally in method 4.

Third, the timing and language style of the extra scenario information delivered by the facilitators affected the pace of the teamwork. Especially, the brief prompts presented in a condensed style as through method 2 and the professional style contributed to the team sustaining a high pace adapted to the targeted clinical tasks. In contrast, the method of delivering information slowly step-by-step, in which the team had to wait for a response to continue, slowed down the teamwork. However, we contend that the varying methods of delivering extra scenario information can serve different learning objectives. The participants’ level of experience and familiarity with simulation as practice are factors to take into account when customizing instructions [8]. Further, the clinical scenario will also make a difference, as simulating the patient’s voice is an option to convey some information in awake patients as opposed to when simulating unconscious patients or infants. A novice team may benefit from facilitation that helps them slow down and learn a procedure step-by-step, while the same type of instruction may disturb an experienced team by deviating from the objective to train them to deliver care under time pressure. Further, the various methods may suit different pedagogical agendas. For example, if educators want to support teams during scenarios, for example, by roleplaying a team member asking a relevant question, the presence of a facilitator is necessary. If another agenda is preferred, in which the participants’ experiences of solving the situation themselves are regarded important for learning, it may be a disadvantage with an instructor in the suit. This points to a need for educators in healthcare simulation to adapt instruction to various demands and learning objectives to ensure high-quality simulation [5, 16, 22].

In sum, the timing of information seemed to be important for the team to sustain engagement, team communication, workflow, and tempo. The language style of the supplied information appeared to have the potential to change the pace, as well as the focus of the scenario. We conclude that interprofessional team training could benefit from these opportunities to optimize the momentum of the workflow.

One methodological limitation was that we did not have access to records of what was said via the earpiece, although we could draw conclusions from the participants’ behaviors. Another limitation was that the analysis relied solely on data from a limited sample of film clips from three Swedish simulation centers. But there were also important strengths. First, that the collaborative analysis of the whole research group enabled the identification of new phenomena and relations, that is, the nature of extra scenario information, how it is conveyed, and its consequences. Secondly, that these findings could be both elaborated and refined by anchoring the initial findings in the whole data set. However, to establish the stability of these phenomena in other settings and the relations between methods and their consequences require a larger data source and hypothesis-driven quantitative studies.

## Conclusions

In this study, we showed the significance of extra scenario information that previous studies largely overlooked. We argue that the communication patterns, workflow, and pace in simulated scenarios are closely related to the method of delivery. We found that facilitators´ close access to teams’ actions enabled timely and brief information that was less disturbing to team communication and the momentum of the workflow compared to instructions given from the adjacent operator room. Finally, the results point to the importance of adjusting in-scenario facilitation to the participants’ level of experience and the learning goals of the session to achieve functional task alignment in healthcare simulations.
